# Evaluating Nanoparticles in Preclinical Research Using Microfluidic Systems

**DOI:** 10.3390/mi10060414

**Published:** 2019-06-21

**Authors:** Derui Zhu, Qifu Long, Yuzhen Xu, Jiangwa Xing

**Affiliations:** 1 Research Center of Basic Medical Sciences, Medical College, Qinghai University, Xining 810016, China; 2007980008@qhu.edu.cn (D.Z.); 2000980001@qhu.edu.cn (Q.L.); 2 Department of Basic Medical Sciences, Medical College, Qinghai University, Xining 810016, China; 2015980003@qhu.edu.cn

**Keywords:** nanoparticles (NP), microfluidics, NP transport, NP uptake, NP accumulation, organ-on-a-chip

## Abstract

Nanoparticles (NPs) have found a wide range of applications in clinical therapeutic and diagnostic fields. However, currently most NPs are still in the preclinical evaluation phase with few approved for clinical use. Microfluidic systems can simulate dynamic fluid flows, chemical gradients, partitioning of multi-organs as well as local microenvironment controls, offering an efficient and cost-effective opportunity to fast screen NPs in physiologically relevant conditions. Here, in this review, we are focusing on summarizing key microfluidic platforms promising to mimic in vivo situations and test the performance of fabricated nanoparticles. Firstly, we summarize the key evaluation parameters of NPs which can affect their delivery efficacy, followed by highlighting the importance of microfluidic-based NP evaluation. Next, we will summarize main microfluidic systems effective in evaluating NP haemocompatibility, transport, uptake and toxicity, targeted accumulation and general efficacy respectively, and discuss the future directions for NP evaluation in microfluidic systems. The combination of nanoparticles and microfluidic technologies could greatly facilitate the development of drug delivery strategies and provide novel treatments and diagnostic techniques for clinically challenging diseases.

## 1. Introduction

With recent advances in nanotechnology, a variety of nanoparticles (NPs) have been fabricated, including liposomes [1,2], gold and silver NPs [3,4], polymeric micelles [5], magnetic NPs [6,7], quantum dots [8,9] and so on. These NPs could vary in size, shape, surface charge and functional groups. By optimizing their properties and screening their performance using in vitro or in vivo systems, these NPs have found a wide range of applications in clinical therapeutic and diagnostic fields [10,11,12,13,14]. NPs could facilitate targeted delivery and control release of loaded chemicals as well as masking out potential side effects of free chemicals [15,16,17]. They can also be used as diagnostic agents for disease diagnosis [18,19].

Currently, despite the fact that several NPs have already been approved for clinical cancer treatment by the US Food and Drug Administration (FDA) and the European Medicines Agency (EMA), including Abraxane^TM^, Doxil^TM^ and gold nanoshells^TM^ [20], more NPs are still in the preclinical evaluation phase. This may be partly due to the difficulties encountered in evaluating NP performance. The human system is highly complex. Conventional static 2D cell culture using one or more cell types is easy and direct to implement, which is often picked as the first model for NP evaluation after it is fabricated. However, it has limited capability in recapitulating actual in vivo microenvironment such as complex cell–cell and cell–matrix interactions as well as chemical and mechanical controls of the cells. The 3D cell culture models such as tumor spheroids could mimic complex tissue structures to a certain extent, but failed to mimic the existence of chemical gradients and flow conditions. As a result, both 2D and 3D cell culture platforms could reach unmatched testing results compared with in vivo [21]. Animal models so far could best evaluate NPs in physiological conditions, however, their inherent inter-species variations in drug and NP responses as well as high cost and long testing periods require more human-relevant models.

On the other hand, the evaluation of NPs is highly phenotype and microenvironment dependent. The delivery of NPs to biotissues such as tumors is a multi-step process. Each step requires different NP design conditions for optimal transport which may conflict with each other, resulting in difficulties to find the best NP design for a targeted delivery [22,23]. At the same time, the patient-dependent phenotypes of biotissues and the heterogeneity of biosamples within the same patient further increase the optimization difficulty [24]. With these challenges mentioned above, better NP testing platforms which could evaluate NPs under physiologically relevant conditions and provide patient-dependent characterizations which are highly needed.

Microfluidic systems can simulate dynamic fluid flows, chemical gradients, partitioning of multi-organs as well as local microenvironment controls, offering an efficient and cost-effective opportunity to fast screen NPs in terms of transport and efficacy for multiple clinical applications [21]. Currently most microfluidic reviews for NP performance testing are focusing on microfluidic techniques and characteristics [20,21,25,26,27], yet to categorize these systems based on their utilities in NP evaluation. Here, we are focusing on summarizing recent microfluidic systems demonstrated to be effective in different aspects of NP evaluations. First, we introduce the major steps of NP delivery and key NP evaluation parameters which are critical for NP performance and highlight the important role that microfluidic systems can play in the testing process. Next, we summarize various microfluidic models currently in development, which are specialized in evaluating NP haemocompatibility, transport, uptake and toxicity, accumulation and general efficacy, respectively. Finally, the challenges and future directions of the development of microfluidic systems for NP evaluation are discussed. 

## 2. Key Evaluation Parameters of NP Efficacy Testing for Drug Delivery 

In clinical practice, a successful delivery of NPs typically involves several steps: (a) intravenous infusion, (b) penetration through blood vessel endothelium, (c) penetration through target biotissue, and (d) uptake and detainment by target cells. During this journey, NPs may face several main problems: rapid plasma clearance, poor penetration, and unexpected organ uptake and accumulation. The plasma clearance is mainly enforced by renal filtration (for NPs < 5.5 nm) [9,28] and macrophage internalization (for NPs of 20 nm–2 μm) [29,30], and physical barriers such as blood vessel endothelium [31,32], blood–brain barrier (BBB) [33,34] and interstitial fluid pressure [35] could further hinder NP transport. Moreover, unexpected NP accumulation in organs such as the liver, spleen and lungs may occur, resulting in fibrotic lesions and toxicity [36,37,38]. In order to attain best NP delivery, many parameters of NPs need to be optimized, such as size, shape, surface charge and functionalization. 

NP size is one of the key factors affecting plasma clearance and accumulation in target tissue. Studies have shown that intermediately sized particles between 30 to 100 nm in diameter might favor longer NP circulation time [39,40], and the phagocytosis rate is highly size-dependent [4,30,41]. At the same, the NP permeability and transport is positively correlated with particle size [42,43,44,45,46]. 

However, these size-dependent effects might be affected or twisted by NP surface charge and functionalization. Charged NPs can be readily opsonized by plasma proteins with opposite charge, ending up in much faster plasma clearance [47,48]. Positively charged macromolecules have faster extravasation property compared with negatively charged ones [43,49,50,51,52,53], and promote cellular internalization [2]. At the same time, neutrally and negatively charged NPs have shown better penetration into tumor tissue compared with positively charged ones [48,51,52,54]. The attainment of a native protein corona for a NP circulated in the blood stream might affect its effective size and surface charge, ending in different plasma half-life and extravasation speed [47,55]. For example, PEGylation, the covalent attachment of one or more polyethylene glycol (PEG) chains, could help NPs of various types to escape plasma clearance [56,57,58]. 

NP shape is another factor affecting target tissue accumulation especially for tumors. It can be optimized for a larger surface area and less travel resistance by choosing spherical asymmetry design such as disk-shape, which could increase NP deposition on vessel walls [59,60]. 

## 3. Positioning of Microfluidic Systems in NP Performance Evaluation 

Currently numerous in vitro models using static culture are being employed to evaluate NP performance. However, one key problem encountered is the interpretation of these testing results with in vivo correlation. Despite the good consistency in some of the scenarios, in vivo–in vitro differences do occur. For example, in vitro studies have shown that silver NPs could exhibit size-dependent cytotoxicity in both macrophages and fibroblasts [61,62]. With smaller NPs, larger cytotoxicity was observed. However, in vivo studies suggested that small NPs tend to be non-cytotoxic [36]. In addition, different testing platforms may result in diverse testing results. Cells cultured in 3D platforms are generally less sensitive to drug treatment compared with those in 2D culture [63,64], the former of which is considered to be more physiologically relevant. These differences raise the need for better evaluation systems with more physiologically relevant environment.

Microfluidics technology features in its precise control of fluids within a submillimeter scale, and offers a variety of applications in both basic and clinical research in the biomedicine field [65,66,67,68]. Compared with 2D and 3D static culture, microfluidic systems exhibit advances in capturing the well-controlled flow pressure in vivo, better spatial and temporal control to the microenvironment, and parallel testing of effects of chemical and oxygen gradient with little cells and chemicals employed (Table 1). Based on its general features, single-cell culture models, complicated 3D tumor cultures and various organs-on-chip models are developed in microfluidic devices [3,69,70,71,72]. They could test NP haemocompatibility, transport, uptake and toxicity, targeted accumulation, and general NP efficacy using different model settings with more physiological relevant testing results.

## 4. Key Microfluidic Models for NP Evaluation 

In this section we will introduce main microfluidic models developed recently for various applications in NP evaluation (Table 2). These microfluidic models mainly use polydimethylsiloxane (PDMS)-glass hybrid setting for biocompatibility and prove-of-concept. Among them, organ-on-a-chip models including various organs and tumors take up the major portion and the rest are mainly single-cell microfluidics or small animal cultures. The development of organ-on-a-chip models has lasted for decades. Through the years, various organs-on-a-chip models have been developed, such as blood vessel-on-a-chip [73,74,75,76,77], lung-on-a-chip [78,79,80,81,82], heart-on-a-chip [83,84,85,86], liver-on-a-chip [87,88,89], kidney-on-a-chip [90,91,92,93], and multiple organs-on-a-chip models [94,95,96], etc. However, not all the models have been applied for NP testing so far. Here we will mainly introduce some of the representative organ-on-a-chip models as well as other types of microfluidic models which have already tested NPs in their systems. With wider applications of existing organ-on-a-chip models and a lot more models in development, an increasing number of microfluidic models would be applied for various aspects of NP evaluation. 

### 4.1. NP Haemocompatibility

**Figure 1:**
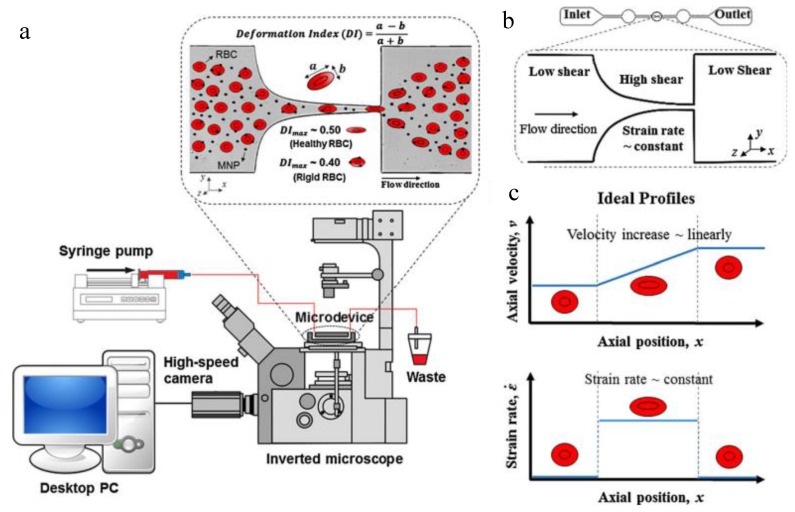
A representative microfluidic device for evaluation the haemocompatibility of NPs (adapted with permission from [103]). (**a**) Experimental setup. (**b**) The geometry of the microchannel device. (**c**) Graphical representation of the fluid-induced condition profiles in the hyperbolic channel.

As discussed earlier, most NPs for clinical uses are intravenously administered into the blood. Therefore, the haemocompatibility of NPs is very critical. Once exposed to blood, NPs need to bring no adverse effects to the blood cells and cause no significant changes in blood plasma [97]. Available in vitro assays evaluating NP haemocompatibility include the hemolysis analysis of red blood cells (RBCs) [97,98,99], leukocyte phagocytosis and inflammation [100], plasmatic coagulation test [101], and platelet activation [98,102]. However, most of these assays are using non-microfluidic experimental setups. Rodrigues et al. recently developed the first microfluidic assay which can detect small rigidity changes of RBCs in the presence of magnetic NPs with much higher sensitivity compared with traditional hemolysis analysis (Figure 1) [103]. The microfluidic channel has a dimension of 20 μm H × 400 μm W × 26,389 μm L, and had a hyperbolic-shaped contraction located at the center of the main channel (Figure 1a,b). This hyperbolic-shaped channel maintains a constant strain rate of about three even when the shear rate changes, and lets RBCs pass through the channel without tumbling and rotations (Figure 1c). Different concentrations of superparamagnetic NPs of 18 nm in diameter were diluted in the Dextran 40 solution (10%, w/v) containing 2% hematocrit (RBCs, v/v) and administered into the microfluidic channel. A high-speed video microscopy system was applied to detect the morphological changes of RBCs at the hyperbolic channel, and the images were analyzed using Image J to calculate the deformation index (DI). Results showed that this microfluidic device could detect the small increase in the rigidity of RBCs when exposed to NPs while the traditional hemolysis analysis showed no significant hematological disorders (hemolysis rates <2%). The increase may be due to the wrapping of NPs by the bilayer membrane of RBCs. This system may help to better understand the biological impact of NPs in clinical applications. 

### 4.2. NP Transport

**Figure 2:**
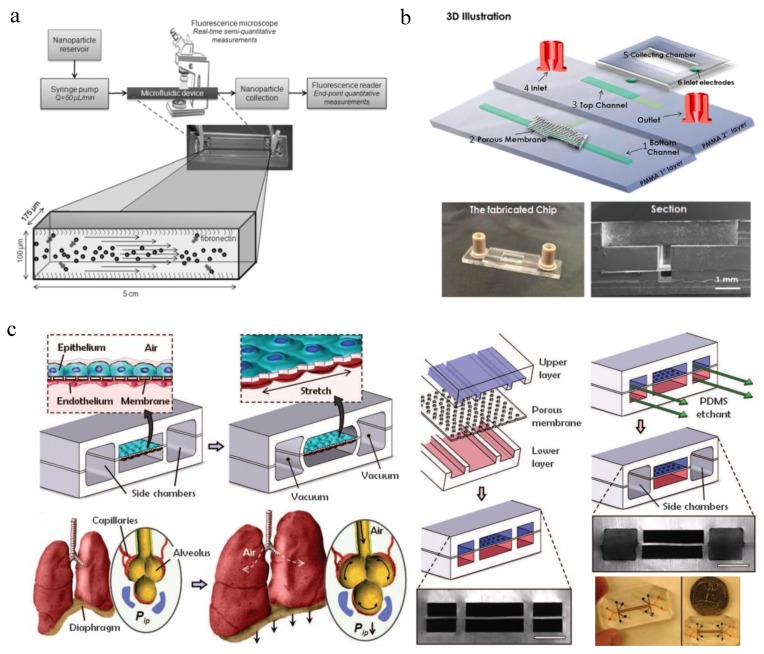
Representative microfluidic systems for evaluation of NP transport. (**a**) Schematic of the cell-free microfluidic system setup for studying the effects of particle size, density and shape on NP margination (adapted with permission from [106]). (**b**) Schematic representation of the blood–brain barrier (BBB) microfluidic system to study NP crossing (adapted with permission from [113]). (**c**) Schematic of a human breathing lung-on-a-chip to study NP transport across the alveolar–capillary barrier (adapted with permission from [82]).

To study NP transport, simple blood vessel-on-a-chip models with or without cells are a good start to mimic NP microcirculation in vivo. These models have been applied to study NP margination [104,105,106], effect of vessel geometry [107] and shear stress [108,109] on NP accumulation, interactions between red blood cells/platelets and NPs [104,110,111] and vessel permeability on NP translocation [31,112]. Figure 2a shows a typical cell-free microfluidic model for studying the margination of NPs during microcirculation [106]. The microfluidic channel was coated with fibronectin and with a dimension of 175 μm W × 100 μm H × 5 cm L, representing the vessel wall. Various NPs including liposomes and metal particles of different sizes, densities and shapes were administered into the channel for eight min at a flow rate of 50 μL·min^−1^ followed by consistent PBS washing for 30 min. Particle margination rates were calculated based on the ratio of the total number of adherent NPs to the sum of the number of adherent and non-adherent NPs. NPs of smaller sizes and oblate shapes had a higher margination rate and deposition percentage on the microchannel wall. More importantly, NPs of lower density deposited significantly more than those of higher density. This study could help find optimal NP design for NP transport and deposition. 

In addition, since blood–brain barrier (BBB) permeability is one of the key ADME properties (i.e., absorption, distribution, metabolism and excretion of chemicals) to be assessed in central nervous system drug discovery, in vitro BBB models are also developed to evaluate NP transport [113,114]. Plenty of non-cell based and cell-based models have been developed so far [115,116], and a comprehensive summary could be found in the recent review [114]. Microfluidic BBB models have been introduced since 2012 [34,71,113,117,118,119,120,121,122,123,124,125], and few models have been applied for NP testing [113]. The microfluidic flow mimics the blood flow in vivo, and a confluent layer of brain endothelial cells was seeded on a transparent polyester porous membrane sandwiched between microfluidic channels on top and below, representing the BBB [113] (Figure 2b). Fluorescent amine-modified polystyrene NPs could transport across the BBB under flow conditions, which could be enhanced when NPs were conjugated with gH625 peptides. 

Other specific organs-on-a-chip models have also been used for evaluating NP transport. For example, lung-on-a-chip systems can study NP transport across the alveolar–capillary barrier [79,82]. In 2010, Huh et al. developed a famous breathing lung-on-a-chip model which could mimic the mechanical distortion of the alveolar–capillary interface during normal breathing [82] (Figure 2c). Human alveolar epithelial cell monolayer and microvascular endothelial cell monolayer were cultured on opposite sides of the extracellular matrix (ECM)-coated PDMS membrane, which could be under cyclic pressure-driven stretching by computer-controlled vacuum and release. When air was administered into the upper epithelial channel, the alveolar cells could maintain viable with increased surfactant production and decreased protein permeability like in vivo. To study NP transport across the alveolar–capillary barrier, NPs of 20 nm in diameter were introduced to the alveolar channel. When no mechanical stretching of the membrane was applied, the translocation efficiency of NPs stayed the same as static transwell experiments. In contrast, when cells were under in vivo-like breathing motions (10% strain at 0.2 Hz), the translocation of NPs into the vascular channel could be significantly increased by four-fold. This mechanical force-induced increase was consistent with in vivo observations and may be due to elevated intracellular reactive oxygen species (ROS) production. 

### 4.3. NP Uptake and Toxicity

**Figure 3:**
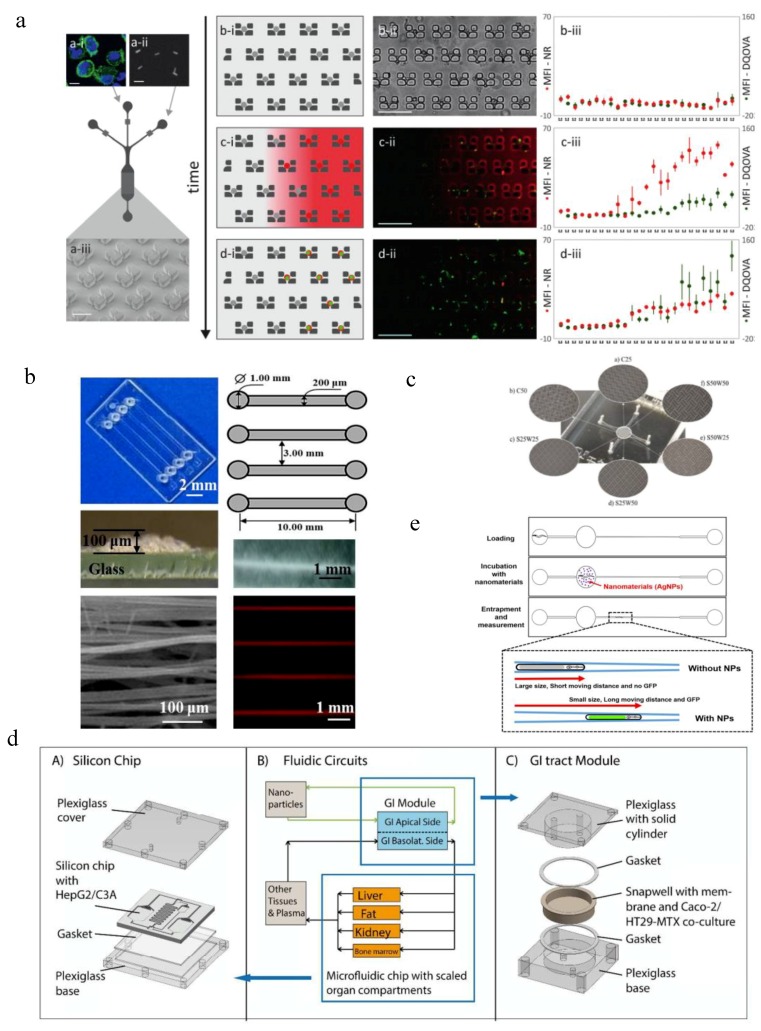
Microfluidic devices for evaluation of NP uptake. (**a**) A single-cell microfluidic system for investigating cell–NP interactions and real-time antigen processing (adapted with permission from [127]). (**b**) Patterned fiber-embedded microfluidic chip to better maintain rat hepatocytes functions and test the hepatotoxicity of Ag NPs (adapted with permission from [136]). (**c**) A microstructured microfluidic platform which could increase medium mixing and reduce NP retention (adapted with permission from [133]). (**d**) The body-on-a-chip system to study the oral uptake of 50 nm carboxylated polystyrene NPs and their induced liver injury (adapted with permission from [72]). (**e**) C. elegans-on-a-chip for evaluating the uptake and nanotoxicity of Ag NPs (adapted with permission from [138]).

Cellular uptake of NPs is quite critical for their clinical applications such as drug delivery and biomarker quantification. Traditional static culture may cause NP sedimentation, the velocity of which will affect cellular uptake efficiency [126]. Microfluidic systems could evaluate NP uptake in perfused conditions. A typical setting could be single-cell microfluidics [3,127]. Cells were seeded into an array of microtraps and exposed to defined concentrations of NPs imposed by microfluidics, and the uptake efficiency was monitored by real-time imaging [127] or cytotoxicity profile [128] (Figure 3a). The advantage of single-cell microfluidics is that it can access the effect of cell heterogeneity [3,128] as well as NP properties [3,127,129] on NP uptake in a high-throughput manner. For example, it has been used to study gold nanorod (NR)-mediated vaccine uptake [127] (Figure 3a). DQ ovalbumin-conjugated gold NRs (NR-DQOVA) was tested using primary bone marrow-derived mouse dendritic cells. The microfluidic device was inserted in a microscope stage incubator for real-time imaging. After the cell was trapped in the microtrap array of 1512 traps, the suspension of NR-DQOVAs and culture medium were delivered from contralateral inlets to form variable NR concentrations across the whole array. Time-lapse microscopy was used to track NR uptake and antigen processing. Cells would become red when in contact with NRs and turn green after antigen processing. Results show the effect of NR concentration on cellular uptake and antigen processing, and the heterogenous cellular responses in single cells when exposed to the same stimuli. Single-cell microfluidics could also study the effect of cell cycle phase on the cytotoxicity of quantum dots (QDs) [128]. HepG2 cells were trapped within the microwells (25 μm in diameter and 20 μm in depth) in the microfluidic device and monitored using a Leica fluorescence microscope. QD solution was administered through microchannels by diffusion to maintain a constant concentration and no shear stress was exposed to the cells. Due to differential cellular uptake efficiency of cells in different phases, the cytotoxicity ranking of QDs is G2/M > S > G0/G1. 

Shear stress could also affect NP uptake by cells [130,131,132]. The efficiency of NP-encapsulated anti-cancer drugs has been shown to be much higher than free drug, both of which could be enhanced when tested in microfluidics. Recently, Mitxelena-Iribarren et al. found that adding microstructures within the microfluidic chamber could increase the medium mixing and reduce NP retention compared with pure laminar flow [133] (Figure 3c). Different sizes of cross shape and linear microstructures with a height of about 30 µm were embedded at the microfluidic chamber. The U-2 OS osteosarcoma cell line was seeded in the chamber as the cancer model. Free methotrexate (MTX), MTX loaded Lecithin-polyvinyl alcohol (PVA) NPs and MTX loaded Lecithin-Tween 80 NPs together with their respective controls were administered to the microfluidic device. The existence of microstructures could slow the cell seeding velocity by 15%–20%, increase the two-dimensional mixing of the flow with an agitation factor higher than 40%, and enhance the vertical distribution of circulating NPs and their interactions with the cells at the device bottom. Cytotoxicity results show that MTX loaded Lecithin-PVA NPs and MTX loaded Lecithin-Tween 80 NPs are much more efficient as promising cancer therapies compared with free MTX, causing cell death of more than 80% and 97%, respectively. More importantly, due to the enhanced cellular uptake of NPs in microstructures containing microfluidic platforms, these cytotoxicity effects could be observed almost 75% faster than those in the plain platform. 

Since liver is the key organ for detoxification and drug metabolism, NP and drug uptake by hepatocytes and Kupffer cells and their hepatotoxicity profiles are also being evaluated [72,134,135,136]. Currently most liver microfluidic models are focusing on recapitulating drug metabolisms and employing cell lines [137], while few assays are trying to test the hepatotoxicity of NPs [8,72] and use primary cells [136]. A patterned fiber-embedded microfluidic chip using primary cells was recently developed to better maintain hepatocytes functions and test the hepatotoxicity of Ag NPs [136] (Figure 3b). Poly-DL-lactide (PLA) fibers with a width of 200 μm and a maximum thickness of 100 μm were coated onto the glass bottom layer of the device, which was bonded to the PDMS top layer with fabricated microchannels. Rat primary hepatocytes were seeded in the chip and cultured under a flow rate of 10 μL/mL. These cells could form 3D spheroids and maintain good hepatocyte functions for at least 15 days as represented by high levels of albumin and urea secretion. When perfused with 120 μg/mL Ag NP solutions at a flow rate of 10 μL/min on day 7 and day 15 for 24 h, the hepatotoxicity profile was quite consistent, both showing about 55% lactate dehydrogenase (LDH) leakage. This patterned fiber-embedded microfluidic liver model was more sensitive to Ag NP-induced hepatotoxicity compared with a normal microfluidic liver model as well as static culture. 

NP-induced liver injury could also be monitored by a body-on-a-chip system to study both NP transport and uptake [72] (Figure 3d). In this model, a coculture of Caco-2 and HT29/MTX cells represents the human intestinal epithelium and was used to study the transport efficiency of 50 nm carboxylated polystyrene NPs; while the HepG2/C3A cell coculture represented the liver and was used to study the NP-induced liver injury indicated by aspartate aminotransferase (AST) levels. Other organs of the human body were also represented. Results showed that about 9.5% of NPs administered could travel across the gastrointestinal barrier and induce the release of AST, indicating liver injury.

Finally, NP uptake and toxicity profile could also be evaluated at small organism level using microfluidics. Currently microfluidic systems have been used to culture various small organisms such as zebrafish [139,140], C. elegans [141,142] and fruit flies [143]. Despite that some of these models have been applied for chemical evaluations [144,145,146], few have been adopted in NP evaluation. C. elegans is one of the model organisms which have been applied for NP testing. The nanotoxicity of silver NPs could be accessed by monitoring changes in C. elegans’ body growth and gene expression [138] (Figure 3e). The PDMS chip fabricated by photolithography was bonded to a glass glide and formed an incubation chamber of 1.5 mm in diameter and a wedge-shaped immobilization channel with the width tapered from 100 μm to 20 μm. Single C. elegans at L4 larval stage was injected into the chip and incubated with/without Ag NPs for either 6 h or 24 h before they were moved to the immobilization channel for imaging and analysis. Results show that the Ag NPs reduce the body length and body width of C. elegans, the latter of which could enable C. elegans to migrate further in the immobilization channel compared with untreated worms. The uptake of Ag NPs could also induce the expression of a metal detoxification protein named metallothionein when detected using a mutant C. elegans strain that express GFP under the control of the mtl-2 promoter, indicating the toxicity of Ag NPs. 

### 4.4. NP Accumulation in Target Tissue

The tumor model is a representative model for studying NP accumulation in target tissue [63,69,147,148,149,150]. The 3D culture configuration in cancer research has shown higher drug resistance and more biomimicry than 2D culture [63,64], and tumor spheroids are currently the most popular models for cancer research due to their 3D configuration, reproducibility and ease of production. Compared with tumor spheroids, microfluidic systems have a much better spatial and temporal control of the culture microenvironment, and form chemical, oxygen and flow pressure gradients to better recapitulate in vivo the NP delivery process. They perform better in studies of angiogenesis and metastasis and are optimal for testing small quantities of drug candidates due to their cost-efficiency. Culturing tumor spheroids in microfluidic configurations could take advantage of the benefits from both systems and make the testing system more biomimicry. It may provide a promising solution to solve the problems of in vivo–in vitro testing differences by better mimicking physiologically relevant testing conditions. 

**Figure 4:**
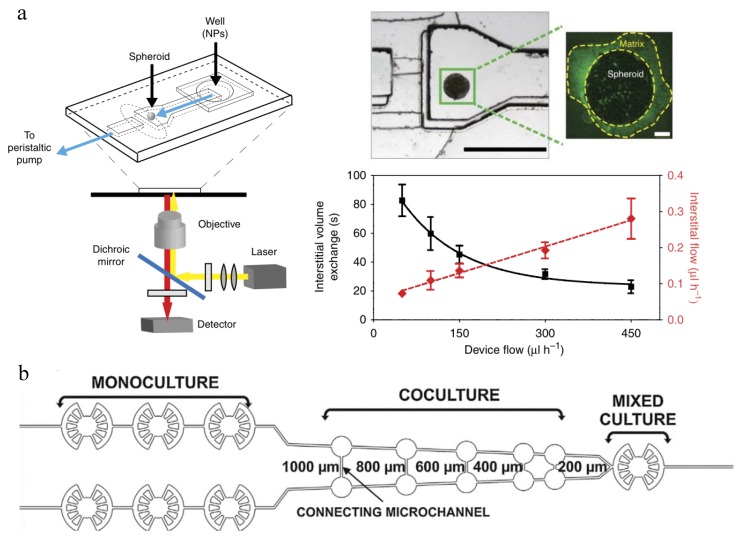
Microfluidic systems for evaluating NP accumulation. (**a**) Schematic and characterization of a tumor-on-a-chip to assess the effect of NP size, receptor targeting and flow rate on NP accumulation in the targeted tumor (adapted with permission from [147]). (**b**) The geometry of a single V-shaped microstructure of the microfluidic system for testing multiple cell culture conditions simultaneously (adapted with permission from [159]).

In vivo-like interstitial flow could be reproduced using tumor spheroids in microfluidics [147,151]. In the model presented by Huang et al. [147], tumor spheroids mimicked the interstitial spaces among cells, the laminin ECM surrounding the spheroids represented the physical barrier between cells and medium, and an optimal flow rate was produced to provide comparable blood flow in tissue capillaries (Figure 4a). This model was used to assess the effect of NP size, receptor targeting and flow rate on NP accumulation in the targeted tumor. Results show that both PEG-conjugated NPs and iron-transporting transferrin (Tf)-functionalized NPs at a diameter of 40 nm could penetrate tumor spheroids and accumulate mostly at interstitial spaces, and the active targeting Tf-NPs had a 15-fold increase in delivery efficiency compared with passive targeting PEG-NPs. When flow rate increased from 50 to 450 μL/h to mimic the variable interstitial flow, both Tf-NPs and PEG-NPs accumulation increased by two folds. However, the accumulation increase was mainly at the tissue interface without deeper penetration, and the NPs would slowly go into the tissue by diffusion. 

Due to enhanced permeability and retention (EPR) effect caused by leaky tumor vasculature and poor drainage from compressed tumor lymphatics, anticancer drugs and even NPs could preferentially accumulate at tumor regions and stay for a longer time compared with normal tissue [152,153,154,155]. However, since tumors are very heterogenous among various tumor types, and many of them have poor vasculature and perfusion, the EPR effect is limited in certain cases [155,156,157]. As a result, only 1%–10% of the anticancer drugs could be delivered to the desired position [28,30,158]. Currently while some efforts have been made to evaluate and avoid unexpected NP accumulation at undesired sites [159,160], few are implemented in vitro [159]. A recent in vitro study was conducted to test the target delivery and toxicity profile of nanoencapsulated photosensitizers for potential photodynamic therapy in cancer treatment [159]. The unique design of the microfluidic device is that it consisted of four V-shaped microstructures which could test monoculture, coculture and mixed culture simultaneously (Figure 4b), and contained a concentration gradient generator that could test four different concentrations at the same time. Normal lung cells (MRC-5) and lung carcinoma cells (A549) were seeded in the PDMS/glass microfluidic device in three types of cultures and exposed to free verteporfin (VP) and polyelectrolyte nanocapsules loaded-VP (nano VP) at a concentration range of 0.25–4 μM. Results showed that VP preferentially accumulated in cancer cells instead of normal lung cells, and low cytotoxicity was observed. Moreover, nano VP exhibited less cytotoxicity than free VP. This microfluidic testing platform could be very useful in testing NPs in different culture configurations under flow conditions. 

As shown above, to date most microfluidic systems are developed to test targeted NP accumulation using single organ-on-a-chip models, thus limiting the ability of testing unexpected NP accumulations in undesired organs or cells. With the advancement of microfluidic technologies and the development of body-on-a-chip models, this bottleneck might be conquered.

### 4.5. NP Efficacy

**Figure 5:**
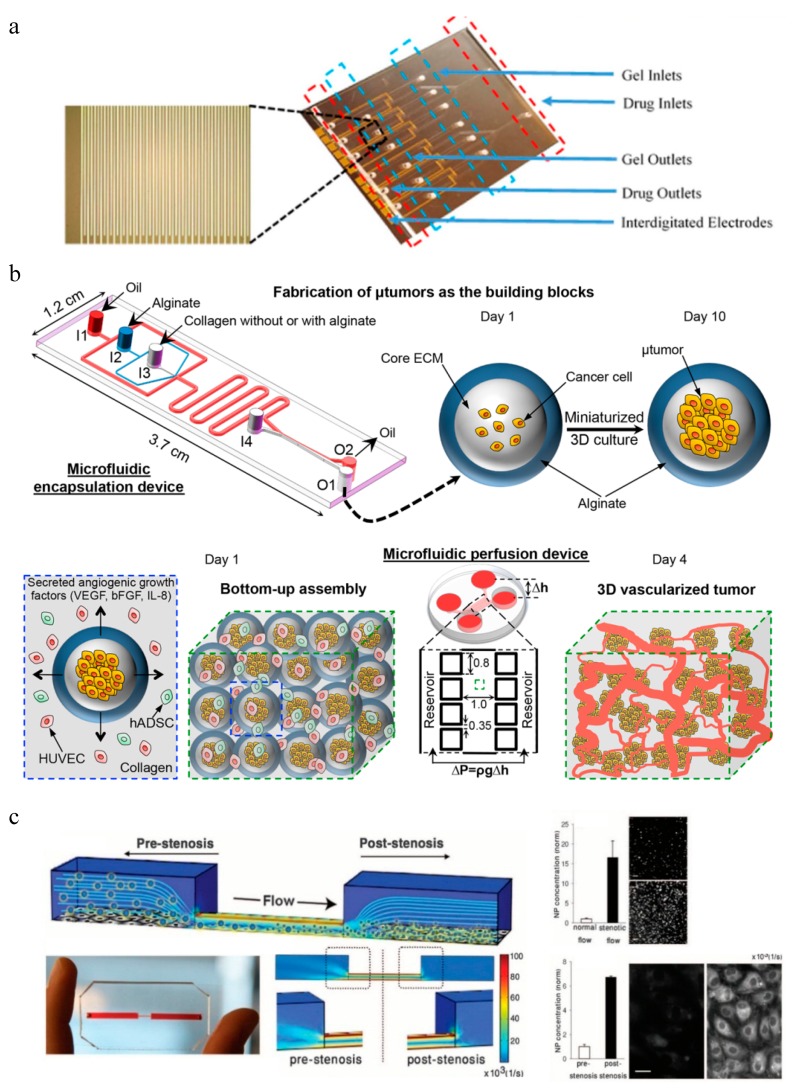
Microfluidic systems for evaluation of NP efficacy. (**a**) Lab-on-a-chip for chemotherapy drug response screening. The interdigitated electrodes are with 10 µm width and spacing (adapted with permission from [161]). (**b**) Schematic illustration of the bottom-up approach for engineering 3D vascularized human tumor model within the microfluidic perfusion device (adapted with permission from [162]). (**c**) A microfluidic vascular stenosis model for shear-induced dissociation of NP aggregated and targeted NP accumulation at stenotic regions (adapted with permission from [163]).

Apart from NP accumulation, tumor-on-a-chip models are also commonly used for evaluation of NP efficacy. For example, integrate microfluidics with electrical microsensors can measure the electrical responses of cancer cells after chemotherapy to fast screen the most effective chemotherapeutic drug within 12 h (Figure 5a) [161]. Cancer cells with different drug responses were seeded separately in the 3D gel structure located in 2 mm × 2 mm chambers with interdigitated microelectrodes, and exposed to parallel microchannels for drug flow. It could differentiate drug susceptible, drug tolerant and drug resistant cells through the change of impedance magnitude which indicated cell viability.

Vascularized 3D tumor models could be created in microfluidic systems to better evaluate NP efficacy. Agarwal et al. recently developed a macroscale 3D vascularized tumor model and evaluated NP-mediated drug delivery (Figure 5b) [162]. They first used a microfluidic encapsulation device to form microtumor capsules. The MCF-7 human mammary cancer cells were encapsulated in core collagen and shelled by alginate, forming a core size and total size of 273 ± 21 μm and 387 ± 15 μm in diameter, respectively. Then these microcapsules were cultured for 10 days for proliferation and stabilization before mixed with human umbilical vein endothelial cells (HUVECs) and human adipose-derived stem cells (hADSCs) in collagen hydrogel and injected into the microfluidic perfusion device. After gelling for 30 min at 37 °C, the whole composite was constrained in the sample chamber by eight micropillars and under perfusion culture for four days induced by hydrostatic pressure. A controlled 3D vascular network with good interconnectedness and lumens could be observed around the microcapsules throughout the whole sample. When testing the efficacy of anticancer drug doxorubicin hydrochloride (DOX) using this microfluidic 3D vascularized tumor model, it showed more drug resistance than both 3D avascular microtumors and cancer cells cultured in 2D. The drug resistance could be reduced by NP-mediated DOX delivery, showing much lower IC_50_ value. This model more mimics the actual microenvironment for tumor progression, invasion, and metastasis, and therefore is more suitable for NP evaluation in terms of anticancer therapy development.

Moreover, microfluidics are unique in detecting the efficacy of shear-activated nanotherapeutics for targeted drug release to obstructed blood vessel areas [163] (Figure 5c). Intact microscale NP aggregates, 1 to 5 mm in diameter, in flow would break up into individual nanoscale NPs when exposed to much higher shear stress at the stenotic area and adhere more efficiently to the blood vessel walls compared with NP aggregates. Therefore, therapeutic agents immobilized on the NPs could be localized at the occlusion or embolism sites, and their therapeutic effects could be evaluated afterwards.

Finally, new microfluidic systems targeting at parallel testing of multiple drugs or cell types towards personalized medicine and treatment are being developed as well. These systems include single-cell-based assays [164], 2D cell monolayers [133,165] and 3D tumor spheroids [166,167,168]. At the same time, by integrating different sensors and imaging systems, microfluidic systems are ready for monitoring NP-induced cell and tissue changes optically, physically and biochemically in a noninvasive way [169]. The advancement in these areas could expedite and facilitate the NP evaluation process, pushing for faster NP development for potential clinical applications.

## 5. Discussion

The organs-on-a-chip model mimicking one or multiple organs is a shining star in recent years and will continue to be one of the major directions for NP evaluation (Table 1). It can take advantage of inherent microfluidic setups and manipulate the microenvironment for biological tissues and organs to provide much better correlation with human pathophysiology compared with static culture models. It has been used for all aspects of NP evaluation, including NP haemocompatibility, transport, uptake, accumulation and general efficacy. The single-cell microfluidic model also has its unique advantages in studying the effects of cell heterogeneity, especially for anti-tumor drug development. With the development of single-cell RNA sequencing [170] as well as other quantitative measuring techniques, single-cell microfluidics may find a wider application in NP evaluation and advance personalized medicine.

Currently the interpretation and translation of NP testing results with microfluidic devices to preclinical and clinical outcomes is still a challenge. One reason may be due to the inherent difficulties in recapitulating in vivo functions of organs using various scaling cultures in current microfluidic cultures, residing in almost all in vitro cell and tissue cultures. Another reason may come from the cell source. Currently cell lines are often used in microfluidic systems instead of human primary cells which are difficult to get and maintain the functions. To mimic in vivo human organ and tissue functions, primary cells rather than cell lines, human cells rather than animal cells or tissue slices are preferred. To overcome this in vivo–in vitro correlation challenge, more sophisticated microfluidic systems may be required, which comes together with a better biological understanding of the systems, higher fabrication costs and more comprehensive system monitoring and measuring techniques. At the same time, to push for wider industrial use, high throughput fabrication and screening compatibility of the microfluidic systems are two important factors as well. A balance between model sophistication and model throughput need to be reached for final industrial adoption. On the other hand, standardized microfluidic systems and protocols for specific NP testing application should also be established, which could make NP testing more efficient and comparisons among different systems possible.

## Figures and Tables

**Table 1:** The main applications, advantages, disadvantages and future directions of microfluidic systems for nanoparticle (NP) evaluation.

NP Evaluation Aspects	Advantages	Disadvantages	Perspectives
NP haemocompatibility NP toxicity NP transport NP uptake by cells Target NP accumulation NP efficacy	Precise spatial and temporal control, which could recapitulate physiological length scales, interstitial flows and concentration gradients Reduce NP sedimentation in static culture by flow control Low cell number and little drug and NP amount required, suitable for personalized treatment and testing Adaptable for real-time imaging and assay-dependent parameter measurements Possible for high-throughput NP testing	Higher cost in terms of chip fabrication More complex in operation Higher chances of getting contamination	Multi-organs-on-a-chip models Single-cell microfluidics High throughput fabrication and screening compatibility Improvement for better in vivo–in vitro correlation System standardization

**Table 2:** Representative microfluidic models for NP evaluation.

NP Evaluation Aspects	Representative Microfluidic Models	References
NP haemocompatibility	Blood vessel-on-a-chip	[103]
NP transport	Blood vessel-on-a-chip	[31,104,105,106,107,108,109,110,111,112]
	Blood–brain barrier (BBB) model	[113]
	Lung-on-a-chip	[79,82]
NP uptake and toxicity	Single-cell microfluidics	[3,127,128,129]
	Tumor-on-a-chip	[133]
	Organs-on-a-chip	[8,72,136]
	Small animal-on-a-chip	[146]
NP accumulation	Tumor-on-a-chip	[144,151,159]
NP efficacy	Tumor-on-a-chip	[161,162]
	Blood vessel-on-a-chip	[163]
